# Protein Level and Infantile Diarrhea in a Postweaning Piglet Model

**DOI:** 10.1155/2020/1937387

**Published:** 2020-05-31

**Authors:** Jing Gao, Jie Yin, Kang Xu, Hui Han, ZeMin Liu, ChenYu Wang, TieJun Li, YuLong Yin

**Affiliations:** ^1^Key Laboratory of Agro-ecological Processes in Subtropical Region, Institute of Subtropical Agriculture, Chinese Academy of Sciences, China; ^2^National Engineering Laboratory for Pollution Control and Waste Utilization in Livestock and Poultry Production, Ministry of Agriculture, Changsha, Hunan 410125, China; ^3^Hunan Provincial Engineering Research Center for Healthy Livestock and Poultry Production, Ministry of Agriculture, Changsha, Hunan 410125, China; ^4^Scientific Observing and Experimental Station of Animal Nutrition and Feed Science in South-Central, Ministry of Agriculture, Changsha, Hunan 410125, China; ^5^University of Chinese Academy of Sciences, Beijing 100039, China; ^6^College of Life Science, Hunan Normal University, Changsha, Hunan, China

## Abstract

Infantile diarrhea is a serious public health problem around worldwide and results in millions of deaths each year. The levels and sources of dietary protein are potential sources of diarrhea, but the relationship between the pathogenesis causes of infantile diarrhea and protein intake remains poorly understood. Many studies have indicated that the key to understanding the relationship between the protein in the diet and the postweaning diarrhea of piglets is to explore the influences of protein sources and levels on the mammalian digestion system. The current study was designed to control diarrhea control by choosing different protein levels in the diet and aimed at providing efficient regulatory measures for infantile diarrhea by controlling the protein levels in diets using a postweaning piglets model. To avoid influences from other protein sources, casein was used as the only protein source in this study. Fourteen piglets (7.98 ± 0.14 kg, weaned at 28 d) were randomly allotted to two dietary treatments: a control group (Cont, containing 17% casein) and a high protein group (HP, containing 30% casein). The experiment lasted for two weeks and all animals were free to eat and drink water ad libitum. The diarrhea score (1 = normal; 3 = watery diarrhea) and growth performance were recorded daily. The results showed that the piglets in HP group had persistent diarrhea during the whole study, while no diarrhea was noticed in the control groups. Also, the feed intake and body weights were reduced in the HP groups compared with the other group (*P* < 0.05). The diarrhea-related mRNA abundances were analyzed by real-time PCR; the results showed that HP treatment markedly decreased the expression of aquaporin (AQP, *P* < 0.05) and the tight junction protein (P<0.05), but increased inflammatory cytokines (*P* < 0.01) than those in control group. In addition, the Adenosine 5′-monophosphate (AMP)-activated protein kinase (AMPK) signaling pathway (*P* < 0.01) was inhibited in the HP group. Intestinal microbiota was tested by 16S sequencing, and we found that the HP group had a low diversity compared the other group. In conclusion, despite being highly digestible, a high casein diet induced postweaning diarrhea and reduced the growth performance of the postweaning piglets. Meanwhile, AQP, tight junction protein, and intestinal immune were compromised. Thus, the mechanism of how a highly digestible protein diet induces diarrhea might be associated with the AMPK signaling pathway and intestinal microbiome.

## 1. Introduction

In many countries, people are still struggling against infection, allergy, and a lack of powerful nutritional control for the infantile diarrhea [1]. Infantile diarrhea is usually distinguished as acute for diarrhea continuing exist for about 2 weeks or acting as a chronic disease if the diarrhea lasts beyond 2 weeks [2]. Diarrhea disease induces high morbidity and mortality in children younger than five years [3]. Generally speaking, gastrointestinal illnesses are a major cause of infantile diarrhea, which induces severe morbidity and mortality among young children. Further, most gastrointestinal illnesses are closely associated with gut microbiota and their products [4]. The composition of the gastrointestinal microbiota is affected by dietary nutrition intake, for example, the three main nutritional components, proteins, carbohydrates, and fats [5]. Protein is the most essential component of tissues in animals and humans, and, unlike carbohydrates, high concentrations of dietary protein intakes could result in several deleterious metabolites in the gut [6]. Amounts of researches that aimed at figure out the effects of protein on infantile and model animals have showed that the concentration and sources of dietary proteins are potential causes of diarrhea in mammals [7]. Members of the aquaporins (AQP) family act as water and ion transporters and complete the rapid transportation of water through membranes in the intestinal tract in the human body; water, via sensitive AQPs, is essential in the migration via diarrhea. While as Zonula occluden-1(ZO-1) and occludin can dynamically affect intercellular permeability. We chose these markers to illustrate the characteristics of diarrhea.

To better understand nutritional control on the development of an individual infantile diarrhea, piglets' model has been chosen as an experimentally analog to human diets. The chosen animal model has been indicated the effects of high protein levels in daily diets on diarrhea, including reduced growth performance, severely watery feces, and gastrointestinal dysfunction. In this work, we have investigated two postweaning piglets' diets that reflected protein's effects on diarrhea. These diets included a control group (Cont, containing 17% casein) and a high protein group (HP, containing 30% casein) that led to an excessive intake of protein compared to that shown in infantile diarrhea. These piglet models provide an approach for clarifying the relationship between infantile protein intake level and its growth performance.

## 2. Methods

### 2.1. Experiment Designed

Our experiment was proceeded in compliance with the Chinese guidelines for animal welfare. Experimental protocol and approved by the Animal Care and Use Committee of the Chinese Academy of Sciences, and the ethical approval code is ISA2017030523.

A total of fourteen piglets weaned piglets (Duroc Landrace Large White, 7.98 ± 0.14 kg, weaned at 28 d) were randomly divided into 2 treatments of 7 replicates each and one piglet one metabolism cage.

Diets (Table 1) according to NRC 2012 were formulated to provide 17% casein protein (CP, containing 17% casein) and a high protein (HP, containing 30% casein), and animals were fed three times per day. The diets were devoid of antibiotics or growth promoters and satisfied all essential amino acids level or exceeded the standard of NRC.

The experiment lasted for two weeks and piglets housed individually in temperature-controlled incubators. All animals were free to diets and drinking water. Piglets were weighed at the starting and ending of the whole test, while feed intake was recorded every day to calculate average daily gain (ADG), average daily feed intake (ADFI), and gain to feed ratio (G: F ratio).

### 2.2. Diarrhea Score

During the whole feeding trial period, diarrhea score (1 = normal; 2 = semiwatery diarrhea; 3 = watery diarrhea) was recorded twice a day (10:00, 16:00) by counting the number of pigs with diarrhea per metabolism cage.

### 2.3. Fecal Fluid Content Determination

When all the piglets of HP group suffered to diarrhea, faeces of each piglet were individually collected in 50 ml centrifuge tube (Sigma), recorded the total weight of each faeces as initiate weight. Collected faeces were baked at 98°C for 3 days, recorded the net weight of faeces. Fecal fluid content was calculated using the formula *W*% = *W*initiate–*W*net.

### 2.4. Slaughter Procedure

Before the end day of the trail, all piglets fasted overnight and were slaughtered by an intravenous injection of sodium pentobarbital (50 mg/kg BW, Sigma) in the last day of the trial.

Blood sampled from the anterior vena cava were collected; serum samples were breakaway form the blood after centrifugation 10 min at 3000 x g and under 4°C. Then, all samples were held at -80°C for analysis.

After slaughtered and cut a midline abdominal incision, gastric, distal ileum, and colon contents were collected. The entire intestinal tract was taking and cut into several segments; 1 cm of ileum was fixed in 4% paraformaldehyde for morphometric analysis.

### 2.5. Gut Morphological Analysis

Fixed tissues were sectioned at 5 mm thickness and stained with haematoxylin and eosin using standard paraffin embedding procedures. 7 intact, well-oriented crypt-villus units were chosen to calculate the ratio of villous height: crypt depth with a light microscope which was loaded with an image analysis system (AxioScope A1, Carl Zeiss, Jena, Germany).

### 2.6. Serum Amino Acids

Serum samples from anterior vena cava were collected for further analysis of amino acid concentration (His, Ser, Arg, Gly, Asp, Glu, Thr, Ala, Pro, Cys, Lys, Tyr, Met, Val, Ile, Leu, Phe, and Trp) by High-speed Amino Acid Analyzer L-8900 (Hitachi, Japan).

### 2.7. Total RNA from Ileum and Real-Time RT-PCR Assays (RT-PCR)

Total RNA from ileum tissue samples was extracted from liquid nitrogen frozen and chop tissues with TRIZOL reagent (Invitrogen, USA), then added in DNase I (Invitrogen, USA) [8] [9]. The reverse transcription was proceeded at 37°C for 15 min, 95°C 5 sec. Primers applied in our experiment were designed by Primer 5.0 according to the pig gene sequence (Table 2). And we chose *β*-actin (the house-keeping gene) to normalize target gene levels. The PCR cycling condition was 36 cycles at 94°C for 40 sec, 60°C for 30 sec, and 72°C for 35 sec. The relative expression was a ratio of the target gene compared to the control gene using the formula 2-(∆∆Ct), where *∆∆*Ct = (Ct Target − Ct *β* − actin)treatment − (Ct Target − Ct *β* − actin) control. Relative expression was normalized and expressed as a ratio to the expression in the control group [10, 11].

### 2.8. 16S rRNA Sequencing and Characterization of Microbiota

Total colon digest genome DNA was extracted by QIAamp DNA Stool Mini Kit according to the manufacturer's instructions, and using sterile water diluted DNA to 1 ng/*μ*L for further study. Distinct region 16SV3-V4 of 16s rRNA genes were amplified used specific primer with index codes on it. All PCR reactions were in the Phusion® High-Fidelity PCR Master Mix (New England Biolabs) condition. PCR products mixed 1X loading buffer (contained SYB green), then used electrophoresis on 2% agarose gel for verification. Picked bright strap between 400 and 450 bp and purified strap with Qiagen Gel Extraction Kit (Qiagen, Germany) for the next experiment.

Used the Ion Plus Fragment Library Kit 48 rxns (Thermo Scientific) following manufacturer's recommendations created sequencing libraries. Then, the library was sequenced on an Ion S5TM XL platform, and 400 bp/600 bp single-end reads were produced. Additionally, the sequencing libraries were appraised by Qubit@ 2.0 Fluorometer (Thermo Scientific) and Agilent Bioanalyzer 2100 system. Raw sequences are available in the NCBI SRA with accession numbers PRJNA600085.

Cut off the low-quality reads by Cutadapt (V1.9.1, http://cutadapt.readthedocs.io/en/stable/), and cut off the barcode and prime sequence to generated paired-end reads [12]. And merged paired-end reads by FLASH (V1.2.7, http://ccb.jhu.edu/software/FLASH/), to avoid the overlap reads generated the opposite end of the same DNA fragment; then, raw tags generated. Using Uparse software (Uparse v7.0.1001) analyzed the high-quality sequences [13]. Sequences were inducted to OTU at ≥97% for further annotation. And taxonomic data was assigned to every representative sequence based on RDP classifier (Version 2.2).

In order to check the phylogenetic relationship of each OTUs and to detect different dominant species between different treatment group, several sequence alignments were proceeded by the MUSCLE software (Version 3.8.31) [14, 15]. OTUs abundance information were conducted by a standard of sequence number parallel to the sample with the least sequences. Alpha (complexity of species diversity) and beta (differences of samples in species complexity) diversity analysis were both conducted according to this output normalized data. To perform alpha diversity, Observed-species, Chao1 (species richness estimator), Shannon (diversity indices), Simpson (diversity indices), ACE (community richness), and Good-coverage (sequencing depth) were calculated with QIIME (Version 1.7.0), and all these indices in our samples were displayed with the R software (Version 2.15.3) [16]. To perform beta diversity, weighted and unweighted unifrac were calculated with QIIME (Version 1.7.0) [16]. Cluster analysis was preceded by principal component analysis (PCA), which was applied to reduce the dimension of the original variables using the FactoMineR package and ggplot2 package in the R software (Version 2.15.3).

Principal Coordinate Analysis (PCoA) was functioned as principal coordinates and visualized from complex, multidimensional data. A distance matrix from weighted or unweighted unifrac calculated result was converted to a new set of orthogonal axes, and basing on the maximum variation factor is demonstrated by the first principal coordinate, and the second maximum one by the second principal coordinate, and persist in the end. PCoA analysis was conducted by WGCNA package, stat packages, and ggplot2 package in the R software (Version 2.15.3). Arithmetic Means (UPGMA) Clustering analysis was used for a type of hierarchical clustering method (to interpret the distance matrix using average linkage) called unweighted pair-group, which is basing on QIME software [17].

### 2.9. Statistical Analysis

Data analysis was done according to the one-way analysis of variance (ANOVA), used Levene's test, and within the student's *t* test (IBM SPSS 21.0 software IIME software (Version 1.7.0)) to make sure the homogeneity of variances.

## 3. Results

### 3.1. Growth Performance, Diarrhea Ratio and Diarrhea Index in Piglets

**Figure 1:**
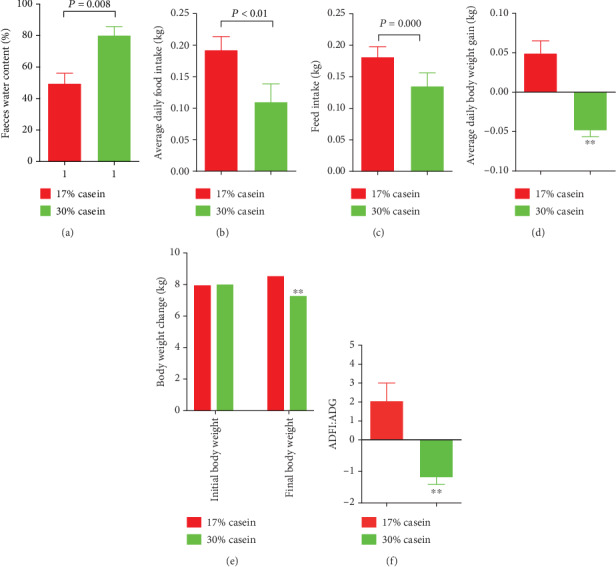
Effects of high casein level diet on the faeces water content and growth performance of piglets. (a) Faeces water content. (b) Body weight change. (c) Feed intake. (d) ADG (average daily gain of body weight). (e) ADFI (average daily feed intake). (f) The ratio of ADFI/ADG. Values are expressed as the mean ± SEM, *n* = 6.

It was showed that high protein level feed could induce persistent diarrhea during the whole study, while no diarrhea was noticed in control piglets(*P* < 0.05) (Table 3) (Figure 1(a)). Also, the body weight (BW) (*P* < 0.05), food intake (FI) (*P* < 0.05), average daily gain (ADG) (*P* < 0.05), average daily feed intake (ADFI) (*P* < 0.05), and feed efficiency (G : F ratio) (*P* < 0.05) were reduced in HP groups compared with the control piglets (Figures 1(b)–1(f)).

### 3.2. Intestinal Morphology

**Figure 2:**
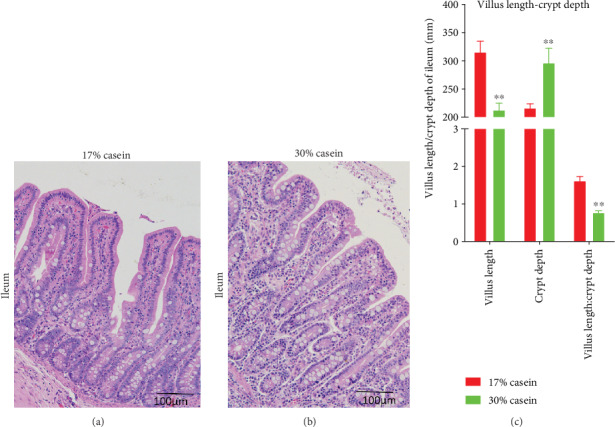
Effects of high casein level diet on morphometrics. Effects of high casein level diet on morphometrics in the ileum in weaning piglets. Representative staining of the ileum mucosal morphology of piglets (magnification: 100x). (a) Ileum morphometrics of control group. (b) Ileum morphometrics of high casein group. (c) Ratio of villus height/crypt depth of control and high casein group. Values are expressed as the mean ± SEM. ∗∗Means the difference was significant (*P* < 0.01), *n* = 6.

The ileum intestinal morphological analysis results of the piglets are shown in Figures 2(a) and 2(b). The height of the villus, depth of the crypt, and the ratio of villus and crypt are shown in Figure 2(c). The results showed that a high protein level diet significantly decreased the height of the ileum villus and increased the depth of the ileum crypt, compared with the control piglets (*P* < 0.05).

### 3.3. Serum Amino Acids

In our experiment, we collected serum samples from anterior vena cava and test amino acid contents, and the result showed that Arg, Gly, Lys, Tyr, Val, Ile, Leu, Thr, Phe, and Trp (*P* < 0.05) significantly reduced in HP piglets compared with the control group (Table 4).

### 3.4. Relative Gene Expression of Tight Junction Proteins

**Figure 3:**
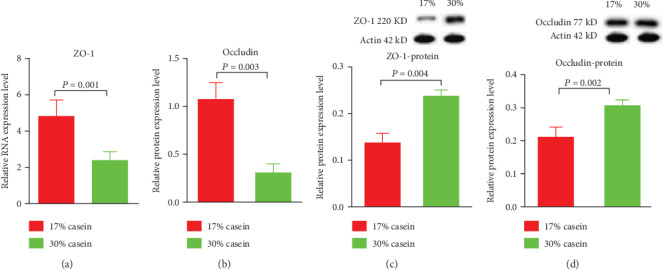
Effects of high casein level diet on the relative gene expression of tight junction protein in ileum. (a) The relative gene expression of ZO-1 in ileum in weaning piglets. (b) The relative gene expression of occludin in ileum in weaning piglets. (c) The protein expression of ZO-1 in ileum in weaning piglets. (d) The protein expression of occludin in ileum in weaning piglets. Values are expressed as the mean ± SEM. *n* = 6.

Provide high casein level feed to piglets decreased the relative expression of ZO-1 and occludin in the ileum (*P* < 0.05), compared with the control group (Figures 3(a) and 3(b)).

### 3.5. Tight Junction Proteins Protein Expressions

ZO-1 and occludin protein expression levels were tested in the high casein piglets and control piglets using Western blot analysis. However, in ileum, the protein expression of ZO-1 and occludin was increased in high casein group piglets compared with the control piglets, which was in contrast to the expression of the mRNA (*P* < 0.05) (Figures 3(c) and 3(d)).

### 3.6. Relative Gene Expression of Aquaporin Protein Family

**Figure 4:**
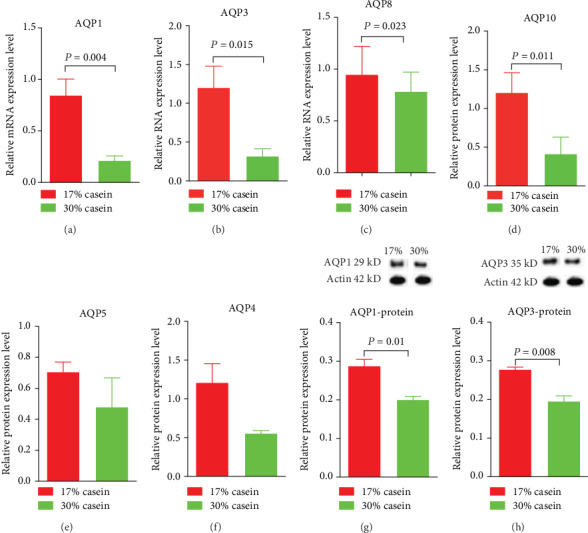
Effects of high casein level diet on the relative gene expression of aquaporins family protein in ileum. (a) The relative gene expression of aquaporins1 (AQP1) in ileum in weaning piglets. (b) The relative gene expression of aquaporins 3 (AQP3) in ileum in weaning piglets. (c) The relative gene expression of aquaporins 8 (AQP8) in ileum in weaning piglets. (d) The relative gene expression of aquaporins 10 (AQP10) in ileum in weaning piglets. (e) The relative gene expression of aquaporins 5 (AQP5) in ileum in weaning piglets. (f) The relative gene expression of aquaporins4 (AQP4) in ileum in weaning piglets. (g) The protein expression of AQP-1 in ileum in weaning piglets. (h) The protein expression of AQP3 in ileum in weaning piglets. Values are expressed as the mean ± SEM. *n* = 6.

Aquaporins (AQP) family were called as water and ion channels which took charge of the rapid transport of water across membranes in the intestinal tract in the human body, and played a key role in the management of water homeostasis [18, 19]. In the intestinal tract, there are several types that are indicated to expressions, such as AQP1, AQP3, AQP4, AQP5, AQP8, and AQP10. And almost all of these AQP in mammalian appear to be highly participated for the transport of water. As expected, we found that the relative mRNA expression of AQP1 (*P* < 0.05), AQP3 (*P* < 0.05), AQP8 (*P* < 0.05), and AQP10 (Figures 4(a)–4(d)) in ileum was strongly decreased in high casein group piglets, while the difference of relative mRNA expression of AQP4 and AQP5 (Figures 4(e) and 4(f)) between HP and cont in ileum was not noticed.

### 3.7. Aquaporins Protein Expressions

Using Western blot analysis, we found the protein expression of AQP1 (*P* < 0.05) and AQP3 (*P* < 0.05) was decreased in high casein group compared with the control group piglets (Figures 4(g) and 4(h)).

### 3.8. Relative Gene Expression of Proinflammatory Cytokines

**Figure 5:**
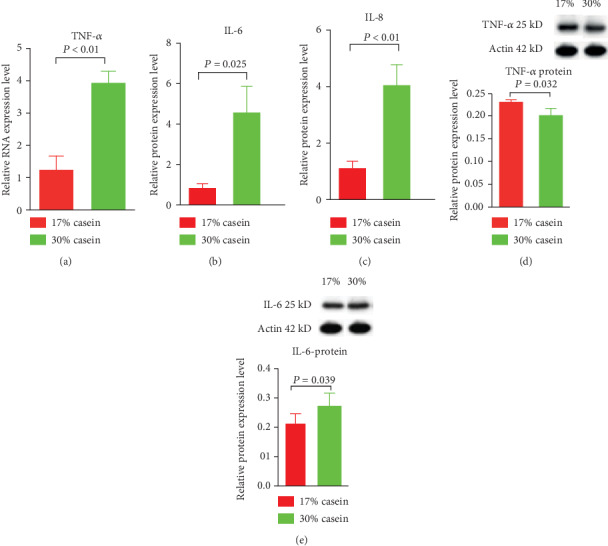
Effects of high casein level diet on the relative gene expression and protein expression of proinflammatory cytokines in ileum. (a) The relative gene expression of TNF*α* in ileum in weaning piglets. (b) The relative gene expression of IL-6 in ileum in weaning piglets. (c) The relative gene expression of IL-8 in ileum in weaning piglets. (d) The protein expression of TNF*α* in ileum in weaning piglets. (e) The protein expression of IL-6 ileum in weaning piglets. Values are expressed as the mean ± SEM. *n* = 6.

When fed high casein level diet to weaned piglets, the relative gene expression of proinflammatory cytokines TNF*α* in ileum were increased (*P* < 0.05); consistent with TNF*α*, we also found that the relative gene expression of IL-6 and IL-8 increased. And using western blot analysis, however, the protein expression of IL-6 increased, whereas the protein expression of TNF*α* decreased (*P* < 0.05) (Figures 5(a)–5(e)).

### 3.9. The Difference in Intestinal Microbiota Diversity and Composition Influenced by High Protein Level

**Figure 6:**
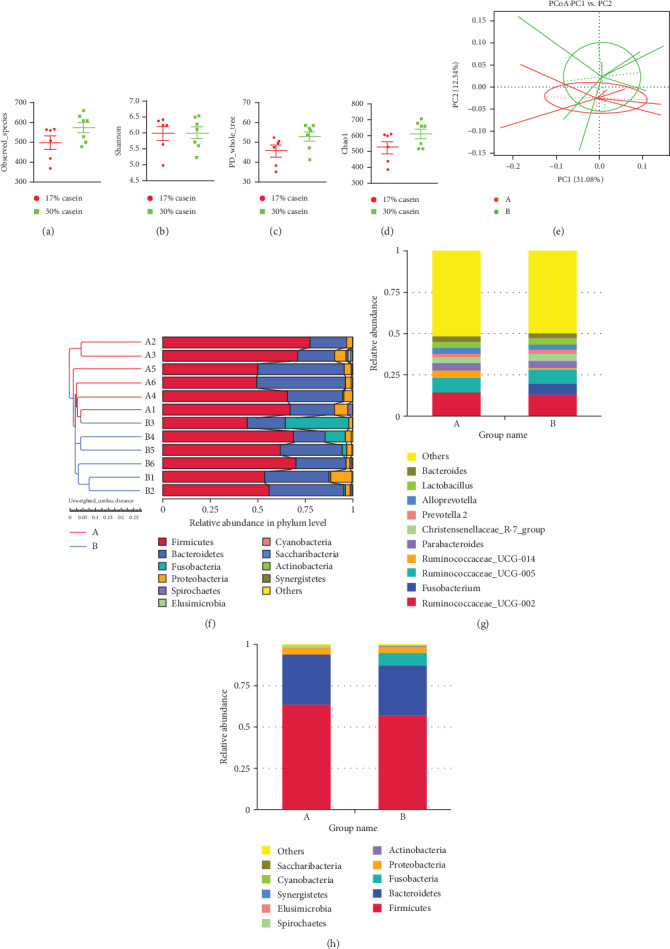
Effects of high casein diet on gut microbial diversity and unweighted UniFrac distances. (a) Observed species. (b) Phylogenetic Diversity (PD). (c) Shannon H index. (d) Chao1 index. (e) Principal Component Analysis (PCoA) of intestinal microbiomes. (f) Comparison of unweighted UniFrac distances between pairs of samples. Data were expressed as the mean ± SEM (*n* = 6); (g, h) 16S RNA bacterial sequences represent in colon samples. A represents control group; B represents high casein diet group. Each figure values of relative abundance of the ten most abundant bacterial groups: genus (g) and phylum (h) found in the colon microbiota (*n* = 6).

The hypervariable V3 and V4 regions of 16S rRNA genes were sequenced from colon content, and about an average of 79,920 ± 5,250 reads were detected by each sample. Picked pairwise identity threshold ≥97%, we got an average of 690 ± 100 operational taxonomic units (OTUs) each sample. Alpha-diversity, including observed-species, Chao1 (species richness estimator), Shannon (diversity indices), Simpson (diversity indices), ACE (community richness), Good-coverage (sequencing depth) were calculated between the microbiomes (Figures 6(a)–6(d)). However, compared to the control piglets, high casein diet seems suppressed the colon microbiota diversity, but the difference showed insignificant (PD, 47.08 ± 3.24 vs. 54.91 ± 2.71, *p* = 0.09; H, 5.98 ± 0.23 vs. 6.0 ± 0.18, *p* = 0.95; chao1, 514.3 ± 38.14 vs. 601 ± 29.78, *p* = 0.1). In order to test the differences between the microbiomes by beta-diversity, we conducted Principal Component Analysis (PCoA), which obtained weighted and unweighted UniFrac distance metric matrices produced for the sample set (Figures 6(e) and 6(f)). And the result of unweighted UniFrac distances in two group piglets indicated that the difference of microbial community structure in two groups showed insignificant (Figure 6).

The total microbial composition for high casein feed showed differences at both the genus and phylum levels (Figures 6(g) and 6(h)). The three largest genera represented in the control dataset *Ruminococcaceae-UCG-002*, *Ruminococcaceae-UCG-005*, and *Ruminococcaceae-UCG-014*, but turned out *Ruminococcaceae-UCG-002*, *Fusobacterium*, and *Ruminococcaceae-UCG-005* in high casein diet piglets, and the difference of Fusobacterium was statistically significant after *t* test (*P* < 0.01). And more importantly, the three largest phyla represented in the control dataset *Firmicutes*, *Bacteroidetes*, and *Proteobacteria*, whereas *Firmicutes*, *Bacteroidetes*, and *Fusobacteria* in high casein group, same with the result in genera level, the high casein diet significant increased colon *Fusobacteria* (*P* < 0.01). The obvious differences were additionally vitrificated by LEfSe analysis, which based on linear discriminant analysis (LDA) to detect bacterial taxa, and it turned out that the sequences are largely abundant in the high casein diet group (data not shown).

## 4. Relative Gene Expression and Protein Expression of AMPK

**Figure 7:**
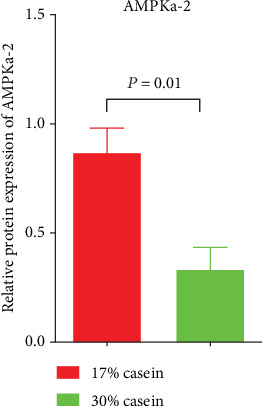
Effects of high casein level diet on the relative gene expression of target of AMPK signal pathway-AMPK*α*-2 in ileum. Values expressed the mean ± SEM. *n* = 6.

According to the real-time RT-PCR assays, high casein level diet decreased the relative gene expression of the target of AMPK signal pathway-AMPK*α* (*P* < 0.05), which indicated that high casein level diet inhibited AMPK signal pathway than low casein diet (Figure 7).

## 5. Discussion

The potential positives of balanced nutrition for the clinical treatment of diarrheal have been researched for many years. To select an appropriate method of nutritional management, the function of the digestive-absorptive metabolism was the first to be studied. Secondly, the reestablishment of the normal physiology of the small intestine needs following with interest.

The high casein level diet of weaning-piglets follows a well-established animal model of postweaning diarrhea. Infantile and early weaned piglets are extremely easy susceptible to intestinal infection according to many factors, resulting in enteric diarrhea. Many parts of the world lace sufficient nutritional control for infantile diarrhea. Because of the immaturity of their digestive systems and immune systems, the weaning period is critical for young humans and swine. When newborns and piglets have diarrhea after being weaned, highly morbidity and mortality were generally occurred [19, 20]. During this period, the composition and level of dietary feeding is a key factor in decreasing the diarrhea incidence [21, 22] (for example, the ingests of the three main nutritional components, proteins, carbohydrates, and fats [5]). Protein is the most essential component of tissues in animals, and, unlike carbohydrates, high level of dietary protein intakes could result in several deleterious metabolites in the gut [22]. Both the severity and incidence chance of diarrhea may be increased in piglets fed diets high in protein level [23].

The maintenance of the normal state of the intestinal epithelium is important to the activities of key physiological processes, such as digestion, absorption, and immune responses. Morphometric results of the small intestine, including the villous height, crypt depth, and the ratio of the villous and crypt, can represent gut health [24]. And increased villous height reflects a larger absorptive area, and a deeper crypt implies a great villous epithelial breakdown, causing some pathogenic inflammation [25]. Previous studies showed that the ratio of villous height and crypt depth was decreased with increased protein dietary level. Further, an early report showed that the intestinal mucosa, given an abundance level of protein, was suffered easily attacked [26, 27] in piglets. In line with the published results, our study shows that the villous height and crypt depth increased, while the ratio of the ileum decreased in high casein group.

Dietary protein is a foundational essential nutrient. Protein levels can influence growth performance and are involved in several pathways, such as the immune system and the assimilation system, in humans and mammalian animals, particularly in elderly adults who need protein to provide nutrition to supporting their growth [11, 28]. After ingestion, dietary protein is hydrolyzed by pepsin and other proteases and formatted into di- and tripeptides and free amino acids (AAs). The imbalanced composition of AA in mammals causes AA antagonism, resulting in reduced food intake and impaired growth [29]. Imbalanced of essential amino acids (EAAs) such as threonine, lysine [30], tyrosine, valine, leucine, glutamine, glycine [31], arginine [32], and tryptophan [33], imbalance in the body induced harmful effects on the health, growth, and development of the animals [34]. In our experiment, we collected serum samples from the anterior vena cava and tested amino acid contents. The results showed that Arg, Gly, Lys, Tyr, Val, Ile, Leu, Thr, Phe, and Trp (*P* < 0.05) were significantly reduced in HP piglets compared with the control group.

Cytokines are closely associated with the immune-inflammatory responses, such as regulating the integrity of the intestinal barrier [35]. Previous studies showed that, in piglets, weaning increased inflammatory cytokines in the intestine. For example, IL-6, IL-8, and TNF*α* could increase the intestinal epithelial, thereby inhibiting the functions of epithelial cells [24, 36, 37]. In our study, a high casein protein level diet increased the ileum's inflammatory responses. IL-6, IL-8, and TNF*α* were significantly increased with the high dietary protein levels, which may partially explain how high protein level diets postweaning diarrhea. Except for the observation that TNF*α* increased protein expression levels, the clear mechanism behind this phenomenon remains unknown. More studies are needed to determine the reasons for the reason.

The present study investigated the nutritional control on the health and development of postweaning diarrhea. Nutrition was shown to replicate the characteristic side effects of high protein level in daily diets on diarrhea including reduced growth performance, and severely watery feces. The high severity and incidence of diarrhea among weaned piglets given the HP diet suggests that high levels of dietary protein triggered postweaning diarrhea, which may be attributed to the fermentation of undigested protein and the dysfunction of tight junction proteins and aquaporins. Importantly, high protein level diets inhibited the AMPK signal pathway, which promotes the incidence and severity of diarrhea. To further illustrate the possible underlying mechanisms of the postweaning diarrhea for a further step, the relative mRNA expression and protein expression of tight junction proteins, which could be regulated by dietary protein, were detected. As we mentioned before, proinflammatory agents can significantly affect intestinal integrity [38]. Our result shows that the expression of IL-6, IL-8, and TNF*α* in the high casein group realized upregulation, thereby launching the intestinal tight junction barrier and enhancing the permeability of intestinal epithelial cells. As reported, the intestinal barrier includes the proteins ZO-1, occludin, and claudin-1 [39]. The increased expression of IL-6, IL-8, and TNF*α* may also affects the expression of ZO-1 and occludin. In our study, with higher dietary casein, IL-6, IL-8, and TNF*α* expression increased in the ileum. Further, the expression of ZO-1 and occludin at the transcriptional level decreased in ileum of piglets, which is consistent with the previous study. To some extent, the abnormal tight junction protein expression could have contributed to postweaning diarrhea [40, 41].

The intestinal epithelium can regulate the absorption of nutrients and fluids, mainly point electrolyte and water absorption and secretion. Diarrhea is closely associated with the disorders of water absorption and secretion [42]. Therefore, to investigate whether a high protein level diet is associated with the potential mechanisms for cellular osmotic homeostasis in a diarrhea animal model, the expression level and potential function of AQPs were determined in our study.

Aquaporins, water, and ion channel proteins have been reported to be of vital importance in water balance. Members of the aquaporins (AQP) family were acted as water and ion transporters and realized the rapid transportation of water through membranes in the intestinal tract in the human body. They are also an integral part of the management of water homeostasis [43]. Several aquaporins exist in the intestinal tract, such as AQP1, AQP3, AQP4, AQP5, AQP6, AQP8, AQP10, and AQP11. Almost all AQPs in mammals seem to be highly discriminating for the transport of water [44]. Moreover, several AQPs have been demonstrated to be downregulated under different types of diarrhea [45].

Accordingly, we found that the expression of AQP1, AQP3, and AQP10 in the ileum was significantly decreased in piglets given a high casein level diet, while the expression of AQP8 was decreased at the mRNA level but remained the same at the protein level. The inhibition of AQP1, AQP3, AQP8, and AQP10 in the ileum has shown the imbalance of water absorption and secretion. With a high casein diet, the expression of some aquaporins becomes inhibited, thereby increasing the fecal water content, and ultimately inducing diarrhea [46, 47]. AQPs played an important role in the transition during diarrhea in many tissues. Among these AQPs, a high protein level diet has a significant influence on the mRNA levels of AQPs in the ileum. Generally speaking, the water transport through the epithelium across the small intestine is regulated by all these AQPs. However, under stress and bacterial infection (e.g., *Fusobacterium*) conditions, AQP1, AQP3, AQP8, and AQP10 are mainly responsible for the maintaining of water homeostasis in the gastrointestinal tract [48]. Also, under a high protein level diet, AQP1, AQP3, AQP8, and AQP10 in the ileum were downregulated, thus interrupting the intestinal permeability during diarrhea. Previous studies have indicated that in the intestine, AQPs act as an alleviator of the dehydration and ions loss by completing a rapid reflow of luminal water back to the body [49]. This activity is possibly explained by the decreased AQP expression in the control group, whose dietary protein level is only 17% higher than that in the high protein group. Importantly, new studies have shown that the intestinal inflammatory response has a striking effect on the inhibition of AQP expression over the postweaning diarrhea period. Therefore, a deeper experimental design is needed to obtain more effective information on the effects of high protein level diets associated with the expression of AQPs and their interactions in the intestine.

The intestinal microbiota serves as an indispensable role in maintaining a host's health by inhibiting the composition of pathogens, supporting the development of a healthy intestinal microbiota, and promoting a beneficial immune system, as well as metabolites for host epithelial cells [50]. In mammals, several studies have showed that production metabolized by the intestinal microbiota somehow affects host metabolism. Piglets are susceptible to various pathogens because of their unstable gut microbiota and immature immune systems [51]. Although the relationship between intestinal microbiota and microbial metabolites in pigs is a research hot spot, we have not confirmed the clear connections, through functional and clinically associated symptoms, between high casein levels and postweaning diarrhea. This is the first study to show a tight correlation between *Fusobacterium* and postweaning diarrhea. *Fusobacterium* is one of the most effective infectious factors that causes intestinal inflammation in piglets. Members of the genus *Fusobacterium* are a group of commensal Gram-negative anaerobes belonging to the phylum *Fusobacteria.* They are usually found in the human oral cavity and are closely associated with periodontal diseases [52]. In our study, we performed a 16S-rRNA gene analysis on colon content samples from all 14 piglets. We discovered that *Fusobacterium* was much richer in the colons of the high casein group piglets than in those of the control group at both the phylum and genus levels. Increasing the abundance of *Fusobacterium* in the colon microbiota seems to act as an important aggravating factor for postweaning diarrhea in piglets. This novel finding may contribute to improved control strategies for infantile diarrhea and postweaning diarrhea in pigs. Clinical studies test on human diarrheal diseases have indicated that *Fusobacterium* can be detected in the colonic tissue of patients within inflammatory bowel disease [53]. And some papers also suggest that *Fusobacterium* is closely associated with the severity of diarrhea in humans and in piglets' model [54, 55]. Species belonging to the *Fusobacterium* genus are closely associated with periodontal diseases. Additionally, some species from the *Fusobacterium* genus act as a cause of colorectal cancer [56]. However, few studies have reported the relationship between *Fusobacterium* genus and diarrhea. Thus, it remains unclear if *Fusobacterium* is induced by the postweaning diarrhea associated with high protein levels in dietary feed. The potential effect of *Fusobacterium* as a pathogenic bacterium has been reported in multiple observations [57]. The specific enrichment of *Fusobacterium* could be due to the following mechanism. The fermentation of undigested protein could engender a failure in the function of the microvilli that grow on the surfaces of intestinal epithelial cells, thereby stimulating the susceptibility to *Fusobacterium* infection. *Fusobacteria* occupy a dominant genus status and are positively populated due to their ability to affix to and invade intestinal cells, thus influencing important function and the metabolic pathways of the gastrointestinal tract, which may induce severe diarrhea in piglet. However, for the time being, this hypothesis remains unsubstantiated.

Recent studies have demonstrated that AMPK participates in the manipulation of water and ion transport, particularly, the expression of transport proteins, including the AQPs and ion transporters in the intestinal epithelium [58, 59]. Importantly, in our experiment, we found that the expression of AMPK was decreased, this may be accompanied by the inhibited expression of aquaporin.

## 6. Conclusion

Our study investigated the nutritional control on the health and development of postweaning diarrhea and indicated that high casein level diet resulted in growth performance, severely watery feces, and a significant diarrhea symptom. And this may be attributed to the fermentation of undigested protein and dysfunction of tight junction protein and aquaporins. Importantly, high protein level diets inhibited AMPK signal pathway, which is promoting the incidence and severity of diarrhea.

## Figures and Tables

**Table 1:** The composition level of basal diet. ∗Premix contained the following per kilogram of the diet: sepiolite, 6.043 g; FeSO_4_ · H_2_O, 516 mg; pig vitamin, 750 mg; MnSO_4_ · H_2_O 250 mg; CoO, 500 mg; ZnSO_4_ · H_2_O, 212 mg; CuSO_4_ · 5H_2_0, 600 mg; Na_2_SeSO_3_, 30 mg; ZnO; VB4 1000 mg.

Diets		
Ingredients	17% casein	30% casein
Casein	19.11	33.72
Corn starch	63.39	45.44
Soybean oil	2	0
Sucrose	5	5
Bran	5	5
Stone power	2	2
Salt	0.5	0.5
Calcium bicarbonate	2	2
Sepiolite	0	5.34
Vitamin-mineral premix	1	1
Calculate analysis		
Crude protein	16.998	29.994
L-Lysine	1.313	2.317
L-(Methionine+cysteine)	0.568	1.001
L-Threonine	0.72	1.271
L-Tryptophane	0.254	0.448
Total energy	15.305	15.387

**Table 2:** Primers used in this experiment. F: forward; R: reverse.

Accession no.	Gene	Primers	Product length (bp)
NM_001167633.1	AMPK1	F: CCTTCGGCAAAGTGAAGGTTG	467
		R: TGCAGCATAGTTGGGTGAGC	
XM_003353439.1	ZO-1	F: GAGGATGGTCACACCGTGGT	169
		R: GGAGGATGCTGTTGTCTCGG	
NM_001163647.1	Occludin	F: TCCTGGGTGTGATGGTGTTC	144
		R: CGTAGAGTCCAGTCACCGCA	
NM_214454.1	AQP1	F: TTGGGCTGAGCATTGCCACGC	221
		R: CAGCGAGTTCAGGCCAAGGGAGTT	
NM_001110172.1	AQP3	F: CACCTCCATGGGCTTCAACT	278
		R: TGCCCATTCGCATCTACTCC	
NM_001110423.1	AQP4	F: CCGGCGGCCTTTATGAGTAT	123
		R: TTCTGTTGTCATCCGCCTCC	
NM_001110424.1	AQP5	F: TGAGTCCGAGGAGGATTGGG	147
		R: GAGGCTTCGCTGTCATCTGTTT	
NM_001113438.1	AQP7	F: AGGCACTTCAGCAGACATCTAA	106
		R: TGGCGTGATCATCTTGGAGG	
NM_001112683.1	AQP8	F: GGTGCCATCAACAAGAAGACG	227
		R: CCGATAAAGAACCTGATGAGCC	
NM_001128454.1	AQP10	F: AGACAGCCTCCATCTTTGCC	212
		R: GTACCCACAGTTGACACCCATG	
NM_001112682.1	AQP11	F: CGTCTTGGAGTTTCTGGCTACC	313
		R: CCTGTCCCTGACGTGATACTTG	
XM_003124280.3	*β*-Actin	F: CTGCGGCATCCACGAAACT	147
		R: AGGGCCGTGATCTCCTTCTG	
NM_001206359.1	GAPDH	F: AAGGAGTAAGAGCCCCTGGA	140
		R: TCTGGGATGGAAACTGGAA	
NM_214399.1	IL-6	F: GCTGCAGTCACAGAACGAGT	118
		R: CAGGTGCCCCAGCTACATTA	
NM_213867.1	IL-8	F: TGCAGAACTTCGATGCCAGT	97
		R: ACAGTGGGGTCCACTCTCAA	
NM_214022.1	TNF-*α*	F: GCCCTTCCACCAACGTTTTC	97
		R: CAAGGGCTCTTGATGGCAGA	

ZO-1: Zonula occluden-1; *β*-Actin: beta-actin; AQP: aquaporins; IL-6: interleukin-6; IL-8: interleukin-8; TNF*α*: tumor necrosis factor alpha.

**Table 3:** The diarrhea rate depending on the diarrhea score.

Index	17% casein	30% casein	SEM	*P* value
Diarrhea rate	13%	96%	0.0812	<0.01

**Table 4:** Effects of high casein level diet on anterior vena cava serum amino acid concentrations in weaning pigs.

Item	17% casein	30% casein
Histone	11.28 ± 1.62	9.08 ± 0.62
Serine	18.49 ± 2.54	13.64 ± 0.56
Arginine	32.36 ± 3.96	21.07 ± 1.07∗
Glycine	66.58 ± 10.90	35.83 ± 2.48∗
Aspartate	9.06 ± 1.28	7.32 ± 0.30
Glutamate	93.45 ± 15.89	59.15 ± 2.72
Threonine	34.05 ± 7.58	5.73 ± 0.58∗
Alanine	48.92 ± 8.63	43.20 ± 2.36
Proline	33.55 ± 7.62	16.86 ± 0.78
Cysteine	3.73 ± 1.14	2.60 ± 0.20
Lysine	44.41 ± 7.42	23.42 ± 0.68∗
Tyrosine	20.29 ± 4.81	6.48 ± 0.44∗
Methionine	5.57 ± 1.09	2.93 ± 0.14
Valine	43.98 ± 7.54	17.85 ± 0.93∗
Isoleucine	23.14 ± 3.57	10.89 ± 0.51∗
Leucine	29.99 ± 4.95	13.86 ± 0.75∗
Phenylalanine	20.51 ± 2.03	13.69 ± 1.72∗
Tryptophan	6.70 ± 1.59	1.03 ± 0.06∗

Values are mg/l. Serum amino acid levels were determined by HPLC Ultimate 3000 and 3200 Q TRAP LC–MS/MS. Data are presented as mean ± SEM, *n* = 6. ∗Within a row, means the difference is significant (*P* < 0.05).

## Data Availability

The data used to support the findings of this study are available from the corresponding author upon request.

## Abbreviations

Cont: Control group
HP: High protein group
AMPK: Adenosine 5′-monophosphate (AMP)-activated protein kinase
BW: Body weight
FI: Food intake
ADG: Average daily gain
ADFI: Average daily feed intake
G:F ratio: Gain: Feed ratio
ENA: Essential amino acids
AQP: Aquaporins
*β*-Actin: Beta-actin
ZO-1: Zonula occluden-1
IL-6: Interleukin-6
IL-8: Interleukin-8
TNF*α*: Tumor necrosis factor alpha.
